# Indigenous engagement in health: lessons from Brazil, Chile, Australia and New Zealand

**DOI:** 10.1186/s12939-020-1149-1

**Published:** 2020-07-31

**Authors:** Angeline Ferdinand, Michelle Lambert, Leny Trad, Leo Pedrana, Yin Paradies, Margaret Kelaher

**Affiliations:** 1 Centre for Health Policy, School of Population and Global Health, University of Melbourne, Parkville, Australia; 2 Ngāi Tahu Māori Health Research Unit, Department of Preventive and Social Medicine, Dunedin School of Medicine, University of Otago, Dunedin, New Zealand; 3 Instituto de Saúde Coletiva, Universidade Federal da Bahia, Salvador, Brazil; 4 Alfred Deakin Institute for Citizenship and Globalisation, Faculty of Arts and Education, Deakin University, Burwood, Australia

**Keywords:** Indigenous, Health, Policy, Chile, Brazil, Australia, New Zealand, Engagement, Participation

## Abstract

**Background:**

Given the persistence of Indigenous health inequities across national contexts, many countries have adopted strategies to improve the health of Indigenous peoples. Governmental recognition of the unique health needs of Indigenous populations is necessary for the development of targeted programs and policies to achieve universal health coverage. At the same time, the participation of Indigenous peoples in decision-making and program and policy design helps to ensure that barriers to health services are appropriately addressed and promotes the rights of Indigenous peoples to self-determination.

Due to similar patterns of Indigenous health and health determinants across borders, there have been calls for greater global collaboration in this field. However, most international studies on Indigenous health policy link Anglo-settler democracies (Canada, Australia, Aotearoa/New Zealand and the United States), despite these countries representing a small fraction of the world’s Indigenous people.

**Aim:**

This paper examines national-level policy in Australia, Brazil, Chile and New Zealand in relation to governmental recognition of differential Indigenous health needs and engagement with Indigenous peoples in health. The paper aims to examine how Indigenous health needs and engagement are addressed in national policy frameworks within each of the countries in order to contribute to the understanding of how to develop pro-equity policies within national health care systems.

**Methods:**

For each country, a review was undertaken of national policies and legislation to support engagement with, and participation of, Indigenous peoples in the identification of their health needs, development of programs and policies to address these needs and which demonstrate governmental recognition of differential Indigenous health needs. Government websites were searched as well as the following databases: Google, OpenGrey, CAB Direct, PubMed, Web of Science and WorldCat.

**Findings:**

Each of the four countries have adopted international agreements regarding the engagement of Indigenous peoples in health. However, there is significant variation in the extent to which the principles laid out in these agreements are reflected in national policy, legislation and practice. Brazil and New Zealand both have established national policies to facilitate engagement. In contrast, national policy to enable engagement is relatively lacking in Australia and Chile. Australia, Brazil and New Zealand each have significant initiatives and policy structures in place to address Indigenous health. However, in Brazil this is not necessarily reflected in practice and although New Zealand has national policies these have been recently reported as insufficient and, in fact, may be contributing to health inequity for Māori. In comparison to the other three countries, Chile has relatively few national initiatives or policies in place to support Indigenous engagement or recognise the distinct health needs of Indigenous communities.

**Conclusions:**

The adoption of international policy frameworks forms an important step in ensuring that Indigenous peoples are able to participate in the formation and implementation of health policy and programs. However, without the relevant principles being reflected in national legislature, international agreements hold little weight. At the same time, while a national legislative framework facilitates the engagement of Indigenous peoples, such policy may not necessarily translate into practice. Developing multi-level approaches that improve cohesion between international policy, national policy and practice in Indigenous engagement in health is therefore vital. Given that each of the four countries demonstrate strengths and weaknesses across this causal chain, cross-country policy examination provides guidance on strengthening these links.

## Background

Global epidemiological research has established a persistent health gap existing between Indigenous and non-Indigenous populations across various national contexts. The available evidence overwhelmingly indicates that Indigenous peoples worldwide have higher morbidity and mortality rates than non-Indigenous people in the same community or area, including in such indicators as life expectancy, adequate child nutrition and infant and maternal mortality [1, 2]. In reflecting on why Indigenous health inequities continue to persist across diverse contexts internationally, King et al (2009) centre the ongoing experiences and impacts of colonisation, globalisation and disrupted ties to land and culture [3]. Loss or weakening of identity has been tied to higher levels of suicide risk [4], mental illness [5] and use of alcohol and other drugs [6, 7] in some Indigenous communities. Chandler and Lalonde’s work in Canada demonstrates that the variation in suicide rates among First Nations youth in British Colombia is strongly associated with the extent to which their communities engaged in practices that fostered cultural continuity [4, 8]. Autonomy and self-determination underpinning Indigenous cultural continuity and strengthened retention of identity are therefore key principles to interrupt pathways to Indigenous ill health and support Indigenous well-being [3].

Within the health care system, mainstream services are often not designed with the health care needs of Indigenous populations at the forefront. This causes a number of barriers preventing Indigenous people from receiving appropriate and accessible health care, contributing to the health gap. Health services may be geographically inaccessible to Indigenous people who live in rural and remote areas [9]. Biomedical services provided may not incorporate Indigenous health values or concepts, such as a holistic approach that recognises connections to land, community and familial ties, mental health and spirituality [3]. Indigenous people may experience racism or discrimination in health care settings, reducing their ability to safely access such services [10–12].

Given the persistence of Indigenous health inequities, many if not all countries with Indigenous populations have developed strategies to improve the health outcomes of Indigenous peoples. Moreover, due to similar patterns of Indigenous health and health determinants across borders, there have been increased calls for greater global collaboration in this field [13]. In the field of Indigenous health much of the comparative literature is centred around Australia, Canada, New Zealand and the United States [14–16], as are many of the professional networks [17, 18], despite these countries representing a small fraction of the world’s Indigenous people [19]. This is primarily due to these four countries sharing a dominant language, a history of settler colonialism and other historical similarities, as well as Anglosphere privileging at a global level.

Latin American countries have demonstrated considerable innovation in developing strategies to improve Indigenous health, alongside a willingness to engage in cross-country learning regarding best practice across the region [20]. However, this level of cross-country transfer has not extended to outside of Latin America. There is therefore a lack of mutual reflections between Latin America and countries outside the region regarding their different strategies developed to support the health and wellbeing of Indigenous peoples. Lixinski (2017) presents the case that Latin America may provide a rich source of knowledge and experience in engagement with Indigenous peoples, particularly for Australia [21]. Lixinski cites the historical reluctance of Canada, Australia, New Zealand, and the United States to engage with international agreements regarding Indigenous peoples such as the United Nations Declaration on the Rights of Indigenous Peoples (UNDRIP) and the International Labour Organisation’s Convention No. 169: Indigenous and Tribal Peoples Convention (ILO 169). This leads to the suggestion that looking towards countries that are more willing to do so, such as in Latin America, may provide relevant lessons. The relative ease and frequency with which countries in the region exchange ideas, policy and best practice regarding Indigenous issues also speaks to the possibility of opening this network to English-speaking countries [21].

This paper considers Australia, Brazil, Chile and New Zealand and how each country approaches the question of Indigenous health. These countries represent a high level of variety with regards to their national contexts (Table 1), Indigenous demographics (Table 2), and approaches to improving Indigenous health and wellbeing.

**Table 1:** Country and health system characteristics

Characteristic	Australia	Brazil	Chile	New Zealand
Economic development				
OECD member country [22]	Yes	Yes	Yes	Yes
Gross national income per capita (US$, 2017) [23]	50,362	15,065	22,170	38,561
Health system				
Percent GDP spent on health (%, 2018 or latest available) [24]	9.3	9.2	8.9	9.3

**Table 2:** Indigenous population characteristics by country

Characteristic	Australia	Brazil	Chile	New Zealand
Percent population Indigenous	2.8 [25]	< 0.5 [26]	13 [27]	16.5 [28]
Indigenous groups/languages	> 100 [29]	> 300 [30]	9 [27]	1
Percent Indigenous population in urban areas	61.4 [25]	35.1 [30]	75.3 [31]	38.9 [32, 33]
Indigenous infant mortality (deaths/1000 live births): ratio of Indigenous to baseline [2]	1.70 (1.55–1.87)	2.65 (2.46–2.86)	1.13 (0.96–1.33)	1.64 (1.45–1.86)

**Table 3:** Governmental recognition of differential Indigenous health needs across the four countries

	Australia	Brazil	Chile	New Zealand
Support for UNDRIP	✓	✓	✓	✓
Signatory to ILO 169	✘	✓	✓	✘
Constitutional recognition of Indigenous peoples and their right to health	✘	✓	✘	✓
National legislation regarding Indigenous health	✘	✓	½	✓
National Indigenous health policy	✓	✓	✘	✓
Has a treaty in place with Indigenous peoples	✘	✘	✘	✓

**Table 4:** Engagement with Indigenous peoples in health across the four countries

	Australia	Brazil	Chile	New Zealand
Establishment of national policy/legislation for engagement with Indigenous peoples	✘	✓	✘	✓
Established mechanisms for shared decision-making and governance	✘	✓	✘	✓

### Indigenous engagement and recognition of Indigenous health needs in national policy

Participation in health is particularly relevant for Indigenous populations, who are often excluded from decision-making and priority-setting processes in the development and implementation of mainstream health services. Indigenous participation involves rectifying this exclusion and reorienting relationships between Indigenous communities and health systems to be more balanced. As such, participation is tied to principles of social, economic and political equity. Moreover, the ability of Indigenous peoples to have control over the health services that serve their communities is underpinned by the principles of Indigenous sovereignty and self-determination.

*Governmental recognition of differential Indigenous health needs* refers to the willingness of governments to acknowledge that Indigenous peoples have different priorities, values and needs in relation to health from the rest of the population. This recognition is necessary for coordinated action on factors that affect Indigenous health and drives practices such as disaggregated data collection, which allows for development and evaluation of targeted programs and policies. *Engagement with Indigenous peoples in health* helps to ensure that health programs and services reflect the values, priorities and contexts of Indigenous communities and enables Indigenous peoples to have control over the factors that affect their health. The principle of Indigenous participation in health and the right of Indigenous peoples to be consulted with in regards to matters that affect them is enshrined in both the UNDRIP and ILO 169 [34, 35]. For countries that have a treaty or treaties in place, this forms the basis for the relationship between the government and Indigenous peoples, including in matters relating to health. This may encompass who has responsibility for Indigenous health, Indigenous health equity and participation and engagement in health [36]. However, in some cases, treaties may run counter to national legislature, creating contradictions and ambiguity [37].

The current article asks to what extent national-level policy in Australia, Brazil, Chile and New Zealand reflects governmental recognition of the health needs of Indigenous peoples, and whether there is policy in place to support engagement with Indigenous peoples in addressing these needs. The article focuses on policy, rather than practice and implementation. There are often significant differences between formal Indigenous health policy and what happens in practice [38, 39]. However, an examination of policy is valuable as it offers insight into how governments understand and respond to their obligations towards Indigenous populations. That is, formal policy illustrates government intentions and provides a set of expectations against which progress can be measured. A national-level focus has been selected for two reasons: first, for ease of comparison and second, because regional-level policy is, on the whole, shaped by the political/governance agenda at the national level.

## Methods

For each country, a review was undertaken of national policies and legislation to support engagement with, and participation of, Indigenous peoples in the identification of their health needs, development of programs and policies to address these needs; and which demonstrate governmental recognition of differential Indigenous health needs. The search strategy was designed to find not only policies and legislation, but also to capture discussion of these in both grey and peer-reviewed literature. Search strategies varied for each country in order to reflect differences in language, correct terminology and names for Indigenous peoples, and in using country-specific databases. However, search terms for each country included ‘Indigenous’, ‘Health’, ‘Engagement’, ‘Participation’, ‘Legislation’ and ‘Policy’ in English. The terms were also used in Spanish for Chile and in Portuguese for Brazil. For each country, government websites were searched as well as the following databases: Google, OpenGrey, CAB Direct, PubMed, Web of Science and WorldCat.

## Results

### Governmental recognition of differential Indigenous health needs

Governmental recognition of Indigenous health needs was demonstrated firstly by the acceptance of international agreements which lay out guidelines for the protection of Indigenous peoples’ rights. Each of the four countries has accepted international frameworks regarding the rights of Indigenous peoples. All four have given their official support of the UNDRIP, and Chile and Brazil are also signatories to ILO 169.

At the national level, governmental recognition was illustrated in a variety of ways across the four countries, including through a treaty, the establishment of national Indigenous health plans, national legislation relating to Indigenous health and the creation of a health care system centred around provision to Indigenous peoples.

#### Brazil

To protect and promote the rights of Indigenous peoples, the National Indian Foundation (FUNAI) of the Ministry of Justice was created in 1967, under the Brazilian military regime. FUNAI established the first organisation of a national health care delivery system for Indigenous groups and continues to contribute to and monitor the healthcare for Indigenous populations currently delivered by the Ministry of Health [40].

During the re-democratisation process of the 1970s and 1980s, against the background of the health reform movement and the pressure of international organisations [41, 42], Indigenous social movements led to the recognition of ethnic and cultural specificity and differentiated social rights in the federal constitution of 1988 (Art. 231, Art. 232, Cap. VIII, Tit. VIII). This included constitutional recognition of the rights of Indigenous peoples to the use of their lands, as well as the protection of Indigenous customs, languages and traditions [43].

In order to reduce cultural barriers for Indigenous people accessing healthcare, legislation was introduced in 1999 for a ‘differentiated but complementary’ healthcare subsystem (SASISUS) [44]. This model is based within the Ministry of Health Special Secretary of Indigenous Health (SESAI) and monitored by a specific health information system (SIASI). The Special Indigenous Subsystem is organised around 34 special Indigenous health districts (DSEIs) which provide differentiated community-level primary healthcare services. Articulation with more complex levels of public healthcare services in urban areas is managed by each DSEI in collaboration with the municipality. In cases where Indigenous patients and their companions need to access health care services far away from the Indigenous villages, the hosting service Indigenous Houses (CASAI), at least one for each DSEI, offers culturally appropriate accommodation.

The National Indigenous Healthcare Policy (PNASPI, 2002) is based on the principle of ‘primary differentiated healthcare,‘ [45] which recognises the cultural specificity of Indigenous communities’ health needs. This policy outlines the articulation between traditional/Indigenous and biomedical/western healthcare knowledges as a strategy to operationalise respect for Indigenous health systems in intercultural contexts as well as the inclusion of Indigenous people in primary health care services. Under this policy, health workforce training is a key strategy to orient public health care practices to the specificities of Indigenous groups [45].

#### New Zealand

There are a number of policies and legislative statues that underpin the New Zealand government's interaction with Māori. The Treaty of Waitangi is the founding document of New Zealand. The Treaty is an agreement, in Māori and English, that was made between the British Crown and the Māori rangatira (chiefs) [36]. Governmental recognition of the distinct health needs of Māori people are encapsulated in three principles that were derived from the Treaty of Waitangi by the Royal Commission on Social Policy and subsequently used to guide government policy [36, 46]. These principles are: *partnership*, or working together with iwi (tribes), hapū (subtribes/kinship groups), whānau (family groups) and Māori communities; *participation*, the involvement of Māori at all levels of decision-making, planning, development and delivery services; and *protection*, which involves the Government working to ensure Māori have at least the same level of health as non-Māori, and safeguarding Māori cultural concepts, values and practices [47, 48]. The New Zealand Public Health and Disability Act 2000 sets out the District Health Boards’ (DHBs) objectives around health inequities and Māori participation in health decision-making [49].

New Zealand has a number of policies that acknowledge the unique health values and priorities of Māori. New Zealand’s Māori Health Strategy, He Korowai Oranga, is based on a holistic vision of Māori health, *pae ora*, and implemented across four pathways for action:

- supporting whānau, hapū, iwi and community development;
- supporting Māori participation at all levels of the health and disability sector;
- ensuring effective health service delivery; and
- working across sectors [50].

He Korowai Oranga is designed to address the New Zealand Health Strategy [51], Objective 11 of the New Zealand Disability Strategy (promote the participation of disabled Māori) [52] and the New Zealand Public Health and Disability Act 2000 [49]. Responsibility for the implementation of He Korowai Oranga is shared among the Ministry of Health and the DHBs and encompasses the whole of the health and disability sector [50].

However, a recently released Waitangi Tribunal report found that New Zealand’s current legislation and health policy (including He Korowai Oranga), primary care policy and strategy frameworks, funding of Māori primary care providers, and monitoring of the primary health sector were not compliant with the Treaty of Waitangi and did not reflect a meaningful commitment to addressing health inequities experienced by Māori [53]. The report addresses claims concerning the way the New Zealand primary care system has been legislated, administered and monitored by the government since the New Zealand Public Health and Disability Act 2000 came into effect. This is the first of a series of reports and investigates whether Māori inequities in health status are the result of the legislative and policy framework of primary care in New Zealand. It recommends that the treaty principles relevant to the provision of health care for Māori should be extended from participation, partnership and protection to include the principles of equity and options [53]. The report found that although the health sector cannot be held wholly responsible and that other sectors are involved in influencing the social determinants of health, the persistent inequitable health outcomes are indicators of health sector-related Treaty breaches resulting from Crown actions, insufficient actions or omissions [53].

#### Australia

In Australia, the Council of Australian Governments (COAG) committed to the 2007 ‘Closing the Gap’ national initiative to eliminate inequities in health outcomes experienced by Aboriginal and Torres Strait Islander people by the year 2030 [54]. The *National Aboriginal and Torres Strait Islander Health Plan 2013–2023* represents a long-term, evidence-based policy framework to underpin the Closing the Gap program [55, 56]. Recognising the strong influence of social determinants of health, the Health Plan takes a broad view of health and centres culture and connection to country in its conceptualisation of Aboriginal and Torres Strait Islander wellbeing [55].

Apart from the National Aboriginal and Torres Strait Islander Health Plan [55], there are a number of specific strategies and policies aimed at addressing Aboriginal and Torres Strait Islander health issues, including the 2015 National Aboriginal and Torres Strait Islander Cancer Framework [57], the National Aboriginal and Torres Strait Islander Suicide Prevention Strategy [58], the Indigenous Australians’ Health Programme [59] and the National Strategic Framework for Aboriginal and Torres Strait Islander Peoples’ Mental Health and Social and Emotional Wellbeing 2017–2023 [60].

Despite a high number of Indigenous health policies and strategies, in 2011, Howse identified a lack of recognition of the specific needs of Aboriginal and Torres Strait Islander people in a review of existing health legislation in Australia [61]. The author reviewed the 69 principal Acts administered by the Commonwealth Department of Health and Ageing, and found that only three specifically refer to Aboriginal and Torres Strait Islander people [61].

#### Chile

In comparison to the other three countries, Chile has relatively few national initiatives or policies in place to protect the health of its Indigenous peoples. Chile is the only country in Latin America that does not have Constitutional recognition of its Indigenous peoples. However, there is legal recognition in the form of the Indigenous Law 19.253, which also created the National Corporation of Indigenous Development, CONADI. Law 20.584, *The rights and responsibilities of the patient in relation to health care* was enacted in 2012 [62]. Article 7 of the law establishes the right of Indigenous peoples to receive culturally appropriate medical attention from public health services situated in areas with high Indigenous populations. In addition, the Special Health and Indigenous Peoples Program (*Programa Especial de Salud y Pueblos Indígenas*, PESPI), situated within the Ministry of Health, aims to contribute to the improvement of Indigenous health, primarily through the development of a model of health that includes an intercultural focus (Table 3).

### Engagement with Indigenous peoples in health

Both the UNDRIP and ILO 169 outline expectations of governments in their treatment and engagement with Indigenous peoples. This includes the right of Indigenous peoples to ‘free, prior and informed consent’ with regards to matters that concern them [34, 35]. Apart from participation in these international frameworks, the four countries exhibited very different ways of conceptualising Indigenous engagement in health in national policy and legislation.

#### Brazil

The PNASPI clearly outlines a number of strategies for Indigenous engagement in the Indigenous public health system. First, the inclusion of two categories of Indigenous community health workers in Multidisciplinary Health Professionals Teams (EMSI), which operate within Indigenous primary health services: Indigenous community worker (AIS) and Indigenous sanitation agent (AISAN). The health program ‘Working with Traditional Midwives’ was established in 2000 in order to provide stronger articulation between traditional Indigenous midwives and primary health services [63].

Secondly, engagement is established through mechanisms for Indigenous participation, labelled ‘indigenous social control’ in the Indigenous health policy. ‘Indigenous social control’ is operationalised by the participation of Indigenous community leaders at different levels of Indigenous health public organisations:

- The Local Indigenous Councils (CLSI), which are consultative and are comprised of local Indigenous councillors named by the Indigenous communities;
- The State Indigenous Councils (CONDISI) for policy evaluation and control as well as deliberative functions. These Councils are composed of 50% Indigenous community leaders, 25% health service managers and 25% health workers;
- The National Forum of the Presidents of State Indigenous Councils (Fórum de Presidentes do CONDISI) at the federal level has consultative attributions and meets every 3 months;
- The Indigenous Health National Conference (CNSI) is a process of consultation which takes place every 4 years. The CNSI delivers health policy recommendations and proposals to the government regarding Indigenous from the local to the national level [44];
- Indigenous representatives also have a seat in the National Health Council (CNS), which can deliberate on health policies.

#### New Zealand

As referenced above, partnership and participation are two of the key principles which encourage government agencies and departments to work in conjunction with iwi and Māori communities with regards to Māori health [47, 64].

The second pathway for action in He Korowai Oranga is Te Ara Tuarua: Māori participation in the health and disability sector. Participation in this instance is largely conceptualised as consisting of the engagement of Māori health service providers and other Māori institutions and having a robust Māori health workforce [64].

In 2015, Statistics New Zealand conducted a study examining the nature and drivers for Crown-Māori engagement [65]. The report found that in recent years, Māori have become more influential stakeholders in both national and local contexts, and that close working relationships between government agencies and Māori have become more common, including in the health sector. This is attributed in part to Whānau Ora, a cross-governmental public sector initiative that aims to support coordination and service delivery across the health, education and social sectors and devolve the delivery of services to community-based commissioning services [65, 66]. Whānau Ora places greater responsibility both on Māori for service delivery and on government to further incorporate approaches that centre Māori communities [65, 66]. Within the Ministry of Health, the report found that key drivers for Māori engagement included Legislation, the introduction of Iwi (tribal) Leader Groups and Treaty settlements that required or supported engagement and Government strategies and programmes [65].

In contrast to this research, the recently released Waitangi Tribunal report into primary care concluded that both the Public Health and Disability Act and the Primary Healthcare framework in New Zealand are in breach of the Treaty of Waitangi [53]. It stated that not enough was being done by government agencies to reduce health inequities for Māori and that the prescribed principles of the Treaty of Waitangi partnership, participation and protection were not considered important by DHBs or other health agencies. In fact, it recommended the addition of the principles of equity and options [53].

#### Australia

The principle of engagement with Aboriginal and Torres Strait Islander people is generally reflected in Australian national policies and strategies. For example, the Aboriginal and Torres Strait Islander Health Plan 2013–2023 takes an approach that builds on the UNDRIP, as well as Aboriginal and Torres Strait Islander community control and engagement, partnership and accountability [55]. While the Closing the Gap initiative was heavily criticised for using a top-down approach and lacking engagement with Aboriginal and Torres Strait Islander peoples [67–69], in 2016 COAG agreed to ‘refresh’ the strategy to reframe focus around a community-led, strengths-based approach [70].

However, the principle of engagement is largely missing from legislation, as referred to previously. Even where the specific needs of Aboriginal and Torres Strait Islander people are represented in the Commonwealth health legislation, mechanisms for shared decision-making and governance are generally not included [61].

#### Chile

In Chile the principles of engagement and consultation of the UNDRIP and ILO 169 are inadequately supported at the level of national legislation and policy. Supreme Decree 66, *Approved regulation governing the indigenous consultation procedure under Article 6 N*^*o*^*1 Letter A) and N*^*o*^*2 Convention N*^*o*^*169 of the International Labour Organization and repealing stated regulations* [71], lays out details of who is entitled to consultation and the mechanisms of such consultation. The Decree is widely criticised for being put in place to limit the rights of Indigenous peoples affirmed in ILO 169 [72] and has led to Chilean legislature being characterised as contradictory regarding Indigenous rights to prior consultation. Intensive nation-wide consultation regarding Article 7 of Law 20.584 was conducted over 2015 and 2016 with the nine Indigenous peoples recognised under the Indigenous Law with the aim to come to a shared understanding regarding the concepts relevant to the rights and responsibilities of patients to culturally appropriate health care [73]. This was the first national consultation undertaken with Indigenous peoples by the Ministry of Health [74]. However, the practical outcomes of this consultation remain uncertain (Table 4).

## Discussion

Overall, examination of these four countries in combination highlights the need for multi-level approaches to ensuring that Indigenous peoples have adequate control and participation in the development of policies and decision-making practices that affect their health. While each of the countries exhibit some strengths in the areas of international policy, national policy and legislation and/or Indigenous health practice, the countries also show a lack of cohesion across these levels. In a cohesive system, the national policy environment would facilitate the operationalisation of principles espoused in international frameworks, which would then be reflected in practice. In contrast, each of the four countries have adopted international agreements regarding the engagement of Indigenous peoples and the recognition of their special status. However, there is significant variation in the extent to which the principles laid out in these agreements are reflected in national policy, legislation and practice.

Brazil and New Zealand both have established national policies to facilitate engagement while such policy is relatively lacking in Australia and Chile. Australia, Brazil and New Zealand each have significant initiatives and policy structures in place to address Indigenous health. However, in Brazil this is not necessarily reflected in practice and in New Zealand the Waitangi Tribunal has found that the existing NZ Public Health and Disability Act, the Primary Care Strategy and Framework, monitoring of the health sector and the funding of Māori primary care providers are in breach of the Treaty of Waitangi. In comparison to the other three countries, Chile has relatively few national initiatives or policies in place to support Indigenous engagement or recognise the distinct health needs of Indigenous communities.

### Coherence between national and international policy

The last few decades have seen a rise in international Indigenous networks and the establishment of international frameworks for conceptualising the rights of Indigenous peoples within the context of their self-determination and cultural and territorial retention. ILO 169 has been instrumental in establishing national and international standards in relation to free, prior and informed consent with Indigenous peoples in relation to matters that concern them. ILO 169 obligates governments to 1) consult with Indigenous peoples ‘through appropriate procedures and in particular through their representative institutions,’ regarding legislative and administrative issues that affect them and 2) allow Indigenous peoples to participate in ‘the formulation, implementation and evaluation of plans and programs for national and regional development which may affect them directly.’ [35] In Article 24, ILO 169 specifically outlines the rights of Indigenous peoples to maintain their health and healing traditions as well as the right to access all social and medical services without discrimination [75, 76]. Similarly, the UNDRIP states that ‘…indigenous peoples have the right to be actively involved in developing and determining health… programmes affecting them and, as far as possible, to administer such programmes through their own institutions.’ [34]

Australia, Brazil, Chile and New Zealand have each lent their formal support to the UNDRIP, and Chile and Brazil have additionally become signatories to ILO 169. The UNDRIP is not legally binding under international law, although it represents the commitments of countries to adhere to the rights codified therein. Upon signing ILO 169, on the other hand, countries agree to legally bind themselves to the obligations expressed. However, the process of ratifying or adopting international agreements is separate from the establishment of domestic legislation that allows for coordinated implementation [77, 78].

The extent to which the principles in these international frameworks have been reflected in national policies regarding recognition of Indigenous health needs and establishment of mechanisms for engagement is variable across the four countries. While Chile and Brazil are both signatories to the legally binding ILO 169, Brazil exhibits the strongest policies for Indigenous recognition, engagement and governance across the comparator countries, while Chile demonstrates the weakest. Although Australia has lent its formal support to the UNDRIP, mechanisms for Indigenous engagement are based on established ethical and practice norms, rather than formal policies or legislation. The basis for Māori engagement rests much more strongly on the Treaty of Waitangi rather than the UNDRIP.

Amongst these case studies, the direct impact of the adoption of international frameworks on domestic policy and legislation is therefore uncertain. However, this is not to say that such frameworks carry no weight. Rather, there is evidence that the establishment of international agreements facilitates the ‘soft transfer’ [79] of agreed-upon principles regarding the treatment of Indigenous peoples which then has a follow-on effect on how Indigenous health is addressed. This transfer may even occur to some extent in cases where an individual country has not adopted a particular international agreement. While Australia is not a signatory to ILO 169, its passage has been influential in the development of key pieces of Indigenous policy. It has been noted that the *Native Title Act 1993* takes on some of the language of ILO 169, in referring to ‘the right to negotiate’ and consultation undertaken in ‘good faith.’ [80] Indeed, in 2003, the International Labour Organization distributed a manual to support the use and understanding of ILO 169, and held up the *Native Title Act 1993* as an example of domestic law that adheres to and supports the principles of ‘rights of ownership and possession of the peoples concerned over the lands which they traditionally occupy’ laid out in the Convention [81].

### Recognition and engagement in national policy and legislation

New Zealand and Brazil demonstrated the strongest policy frameworks with regards to governmental recognition of Indigenous health needs and the engagement of Indigenous peoples in health. These two countries recognise Indigenous peoples and their right to health and health care and have established mechanisms for shared decision-making and governance, supported by national policies or legislation for Indigenous engagement; however, in both countries these frameworks have not been successfully implemented.

An international comparison of legislative approaches to Indigenous health found that Constitutional recognition of Indigenous peoples and their special status helped to form the basis of coherent national Indigenous health legislation in the cases of New Zealand and the United States [61]. This is important in creating congruence both between international and national frameworks and within the country’s jurisdictional levels. The current case studies uphold this pattern, which suggests that Constitutional recognition may engender the creation of legislation to facilitate Indigenous engagement and thereby protect Indigenous peoples’ rights.

New Zealand’s Treaty of Waitangi between Māori and the British Crown is often recognised as having a major influence in the establishment of legal recognition of Māori peoples and in establishing obligations of the Government with respect to Māori sovereignty. Principles derived from the treaty were used to support the incorporation of Māori health needs in national legislation through the *New Zealand Public Health and Disability Act 2000*. In addition, this legislation outlines mechanisms by which the health needs of Māori peoples are to be determined and addressed, including through community participatory processes [61, 82]. While this legislation has not been implemented successfully and has recently been recognised as a breach of Treaty obligations [53], the case of New Zealand illustrates the utility of Constitutional recognition and an established treaty in the development of Indigenous health policy. In contrast, the cases of Australia and Canada, which do not have Constitutional recognition, exhibited weaker and more piecemeal legislative structures [61].

In Brazil, the right to health for all people was enshrined under the 1988 Constitution, as well as the rights of Indigenous peoples to the use of their lands and retention of their languages and cultures [43]. Subsequently, the differentiated health care subsystem for Indigenous people and the cultural right to health care were born within the national health care reform movement [83]. Despite the support of international instruments ILO 169 and the UNDRIP, Chile’s national legislative and policy framework is inadequate to support sustained engagement and shared decision-making with Indigenous peoples regarding their health needs. Of the four countries, Chile has the weakest national infrastructure in this regard. Of note, Chile is one of only two countries in Latin America that does not have Constitutional recognition of its Indigenous peoples or their rights [84].

### Distinctions between policy and practice

The purpose of this study was to analyse national-level policy and legislation, rather than taking into account policy implementation or real-world practice. However, it is important to note that the difference between Indigenous health policy and practice can be substantial.

Of the four countries, Brazil exhibited the strongest national frameworks regarding recognition of Indigenous health needs and Indigenous engagement; however, the Indigenous health subsystem has been heavily and consistently criticised for a lack of accountability to and representativity of the Indigenous communities, as well as failing to ensure that decisions made through such processes are implemented. From its establishment, the SASISUS presented management problems and outsourcing of Indigenous health care services and delivery to non-governmental organisations, low levels of quality and coverage [38, 85], as well as an inefficient monitoring information system of Indigenous health conditions [86]. While the concept of interculturality or cultural appropriateness forms the backbone of Brazil’s Indigenous health policy, including the differentiated health care model and the PNASPI, there is a lack of clarity around the term, which has also been identified as being ethnocentric [87, 88]. In this regard, the SASISUS model was established to increase inclusivity of the health care system, but remains operationally normative [87] and reproduces the hegemony of biomedical knowledge in health care practices for Indigenous people [89, 90].

As can be seen, New Zealand has a strong basis for the engagement of Māori people in health and for the recognition of Māori values, priorities and needs in the form of the Treaty of Waitangi. However, there has been some indication that engagement with and recognition of Māori people is being dismantled. In 2006, senior management in the Ministry of Health instructed staff to remove all references to the Treaty from health policy [91, 92]. Following this, a review of 49 public health strategies and plans published between 2006 and 2016 found that none of the documents made mention of the Māori text of the treaty and 75% made no reference to the English version [91]. Given the importance of the treaty in establishing Crown obligations to protect Māori health and affirm Māori sovereignty, its implicit rejection in the development of health policies signals a reluctance to engage with these principles. Moreover, the Waitangi Tribunal inquiry and subsequent first report into Primary Care for Māori has outlined the deficits in government policies in relation to reducing health inequities and adhering to their Treaty of Waitangi obligations [53]. This inquiry also found that the government did not collect sufficient quantitative or qualitative data to inform decisions about health policy for Māori and as a result these inequities persisted without a clear understanding of the problems underlying Māori health inequity. A failure to monitor performance of primary health sector with regard to Māori health also contributes to this inequity [53].

In Australia, the situation is rather different--despite the lack of formal national policy or legislation to this effect, various mechanisms do exist to support Aboriginal and Torres Strait Islander engagement at multiple levels of policy and service development. In particular, the Aboriginal community controlled health care sector is recognised as essential to not only health care provision, but in shaping Indigenous health policy and research [93, 94]. Nevertheless, these mechanism structures are generally not based in legislation and fall short of shared decision-making responsibilities. Rather, the impetus for Aboriginal and Torres Strait Islander engagement relies on established norms and ethics regarding practice in Aboriginal and Torres Strait Islander health [95].

### Limitations of the study

There are three main limitations to this study. First, this research considers only policy as it exists on paper, and does not fully consider any discrepancies that may exist between the written policy and real-world implementation. While this decision was made for ease of comparison, it is acknowledged that in some cases this gap is significant. Secondly, we present here only national-level policy, not local or regional. Particularly in Brazil, New Zealand and Australia, a large proportion of the responsibility for Indigenous health policy is held at regional levels. The policies presented here are therefore an underrepresentation of the full Indigenous health policy environment. Finally, while significant effort was made to ensure all relevant pieces of policy and legislation were identified within the four countries, the study is not a systematic review. It may therefore be that some elements of national-level Indigenous health policy in the comparator countries have inadvertently been missed.

## Conclusions

In recent years, there has been an increased trans-national influence on Indigenous policy, bolstered by the recognition that Indigenous peoples worldwide are facing similar challenges and hold similar goals with respect to health. The creation of international policy frameworks has been important in shaping a shared understanding that Indigenous peoples have the right to participate in the formation and implementation of health policy and programs to address their health needs. However, the adoption of international agreements does not necessarily directly lead to the relevant principles being reflected in national legislature and policy, although it may reflect the ‘soft transfer’ of those ideas and standards. At the same time, while a national legislative and policy framework may be created to support the engagement of Indigenous peoples, this may not necessarily translate into practice. Developing approaches that improve cohesion between international policy, national policy and practice in Indigenous engagement in health is therefore vital. The inclusion of countries outside of Australia, Canada, New Zealand and the United States in cross-country comparative Indigenous health research may be useful in providing guidance to strengthen these links.

## Data Availability

Not applicable.
